# A Mobile- and Web-Based Health Intervention Program for Diabetes and Prediabetes Self-Management (BetaMe/Melon): Process Evaluation Following a Randomized Controlled Trial

**DOI:** 10.2196/19150

**Published:** 2020-12-01

**Authors:** Virginia Signal, Melissa McLeod, James Stanley, Jeannine Stairmand, Nitin Sukumaran, Donna-Marie Thompson, Kelly Henderson, Cheryl Davies, Jeremy Krebs, Anthony Dowell, Rebecca Grainger, Diana Sarfati

**Affiliations:** 1 Department of Public Health University of Otago Wellington Wellington New Zealand; 2 Biostatistical Group, Dean's Department University of Otago Wellington Wellington New Zealand; 3 School of Medicine University of Otago Dunedin New Zealand; 4 Kokiri Marae Wellington New Zealand; 5 Department of Medicine University of Otago Wellington Wellington New Zealand; 6 Department of Primary Health Care and General Practice University of Otago Wellington Wellington New Zealand

**Keywords:** diabetes mellitus, prediabetes, self-management, eHealth, mobile apps, evaluation, diabetes, digital health, app, utilization, user perception, user

## Abstract

**Background:**

Technology-assisted self-management programs are increasingly recommended to patients with long-term conditions such as diabetes. However, there are a number of personal and external factors that affect patients’ abilities to engage with and effectively utilize such programs. A randomized controlled trial of a multi-modal online program for diabetes self-management (BetaMe/Melon) was conducted in a primary care setting, and a process evaluation was completed at the end of the study period.

**Objective:**

This process evaluation aimed to examine the utilization patterns of BetaMe/Melon, identify which components participants found most (and least) useful, and identify areas of future improvement.

**Methods:**

Process evaluation data were collected for intervention arm participants from 3 sources: (1) the mobile/web platform (to identify key usage patterns over the 16-week core program), (2) an online questionnaire completed during the final study assessment, and (3) interviews conducted with a subset of participants following the study period. Participants were classified as “actively engaged” if any usage data was recorded for the participant (in any week), and patterns were reported by age, gender, ethnicity, and diabetes/prediabetes status. The online questionnaire asked participants about the usefulness of the program and whether they would recommend BetaMe/Melon to others according to a 5-point Likert Scale. Of 23 invited participants, 18 participated in a digitally recorded, semistructured telephone interview. Interview data were thematically analyzed.

**Results:**

Out of the 215 participants, 198 (92%) received an initial health coaching session, and 160 (74%) were actively engaged with the program at some point during the 16-week core program. Engagement varied by demographic, with women, younger participants, and ethnic majority populations having higher rates of engagement. Usage steadily declined from 50% at Week 0 to 23% at Week 15. Participants ranked component usefulness as education resources (63.7%), health coaches (59.2%), goal tracking (48.8%), and online peer support (42.1%). Although 53% agreed that the program was easy to use, 64% would recommend the program to others. Interview participants found BetaMe/Melon useful overall, with most identifying beneficial outcomes such as increased knowledge, behavioral changes, and weight loss. Barriers to engagement were program functionality, internet connectivity, incomplete delivery of all program components, and participant motivation. Participants suggested a range of improvements to the BetaMe/Melon program.

**Conclusions:**

The program was generally well received by participants; active engagement was initially high, although it declined steadily. Maintaining participant engagement over time, individualizing programs, and addressing technical barriers are important to maximize potential health benefits from online diabetes self-management programs.

**Trial Registration:**

Australian New Zealand Clinical Trial Registry ACTRN12617000549325; https://tinyurl.com/y622b27q

## Introduction

Long-term conditions pose a great burden to patients and health services [1,2]. Furthermore, the economic burden on health systems is growing globally [3-6]. Self-management interventions are a potential way to address this burden, with evidence that such interventions can effectively improve users’ physical and mental health [7]. Digital health interventions are increasingly available to support patient self-management [8-10]. However, there are personal and external factors that affect a person’s ability to engage with and effectively utilize digital health interventions. These include age, motivation, personal values, lifestyle, digital literacy, and support from family and peers [11,12]. Factors external to an individual that influence engagement include the quality of the digital health intervention itself, internet access, level of support provided to enroll and participate, cost to the participant, clinical endorsement, and participant perceptions of data safety [11,12]. The degree to which programs have been designed using contextually relevant models and theory that target the desired behavior (eg, the Behaviour Change Wheel [13]) may be important to outcomes [14].

The BetaMe/Melon digital health intervention is a comprehensive mobile and web-based technology program for people with type 2 diabetes or prediabetes that uses principles of behavioral change theory to support and enhance users’ self-management techniques [15]. The program was developed in partnership with primary care practitioners, Māori and Pacific health providers, and psychologists, and piloted with people with prediabetes [15]. The 12-month program has an initial 16-week active support phase comprising four intervention components: (1) health coaching (during which the first session with the health coach is the only compulsory component of the program), (2) goal setting and tracking, (3) online peer support in a secure forum, and (4) provision of evidence-based resources. The remaining 36 weeks use online peer support and goal tracking only.

Although multimodal digital health intervention programs such as BetaMe/Melon have been shown to be effective in supporting users’ glycemic control [16-19], there are still many knowledge gaps regarding the use and efficacy of digital health interventions. For example, little is known about the mechanisms that might enhance individual engagement and thus have the greatest impact in terms of utilization and adherence [11,12,20,21]. There is also a lack of high-quality empirical evidence as to how individuals incorporate digital health intervention programs into their daily lives, which program components they find most helpful, and how they feel such programs could be improved [9,21].

Process evaluation is an essential part of assessing complex interventions [22,23] and can provide information about how an intervention might work in a given context, factors that may have impacted an intervention’s outcomes, and modifiable factors that might improve an intervention’s success [22]. The aims of our process evaluation were to assess how consistently and in what ways participants used the BetaMe/Melon mobile/web platform and to identify which program components participants found to be the most (and least) useful so as to identify future areas of improvement for this and other similar digital health intervention programs.

## Methods

### Design

We undertook a randomized controlled trial (RCT) of the BetaMe/Melon program with the design and methods described elsewhere [15]. Briefly, the RCT was carried out from June 2016 to June 2018 and compared the outcomes for people aged 18-75 years with hemoglobin A_1c_ (HbA_1c_) of 41-70 mmol/mol enrolled in the BetaMe/Melon program or receiving usual primary health care. The RCT recruited 429 participants, with half (n=215) randomly allocated to the intervention arm (BetaMe/Melon) and half (n=214) allocated to the control arm (usual primary health care) of the study. The primary outcomes were mean changes in HbA_1c_ and weight from baseline to 12 months. A number of secondary outcomes were also measured [15].

**Figure 1:**
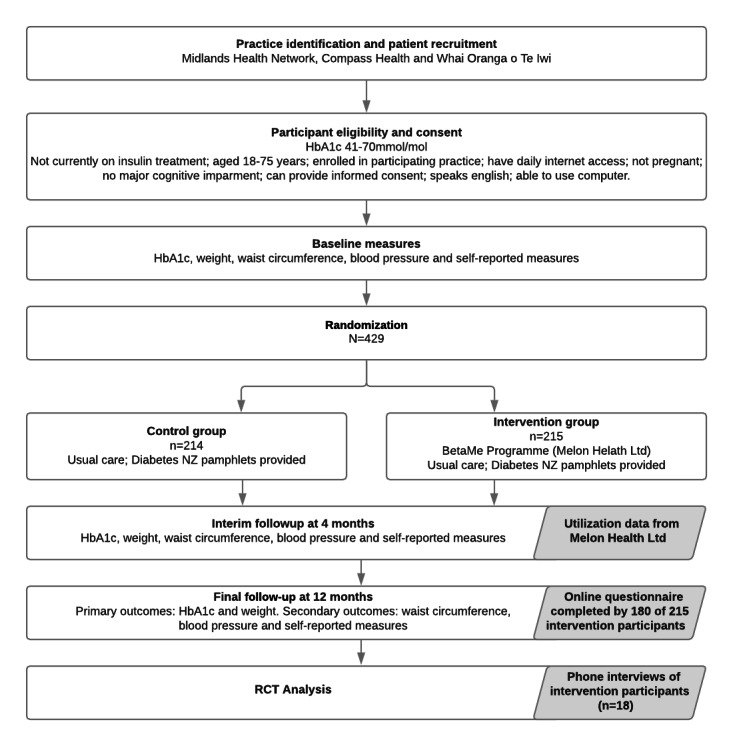
Study flow diagram. HbA_1C_: hemoglobin A_1C_; RCT: randomized controlled trial.

Process evaluation data were collected for intervention arm participants from three sources: (1) the mobile/web platform (to identify key usage patterns during the 16-week active support phase), (2) an online questionnaire completed during the final study assessment, and (3) semistructured telephone interviews conducted with a subset of participants at the end of the study period. A study flow diagram highlighting when data were collected and analyses conducted is provided (Figure 1).

### Ethical Approval

Ethical approval was given by the New Zealand Health and Disability Ethics Committee (approval reference: HDEC 17/CEN/49). The process evaluation was conducted by researchers from the University of Otago. All design, implementation, analytic, and dissemination aspects of the RCT and this process evaluation were the sole responsibility of the Otago research team. The research team had no financial relationship with the company (Melon Health Ltd) who developed the BetaMe/Melon program and delivered the program for the study under contract to the University.

Participants gave written consent to participate in the RCT, which included the use of information from the mobile/web platform to evaluate the BetaMe/Melon program. Additional verbal consent was gained from those participating in the telephone interviews at the end of the study period.

### Utilization Patterns

Online usage data were provided by Melon Health to the University of Otago research team for analysis. The provided dataset covered the program’s first 16 weeks (active support phase) and included data on the first health coach session and all events where participants made an online diary entry or actively engaged with the peer support forum (posted, replied to, or “liked” messages). No data were available on passive interactions with the online program (eg, reading forum messages without commenting or liking) or use of educational resources.

For the utilization analysis, usage data were linked to demographic data collected during the baseline study assessment of the RCT. Participants were classified as “actively engaged” if any usage data was recorded for the participant (in any week), while participants with no usage data recorded were classified as “not engaged” during the active support phase. Proportions engaged are reported with 95% CIs, with differences between key demographic and clinical groups calculated as absolute differences with 95% CIs (differences not adjusted for other variables). Weekly engagement with the online program components was assessed for the active support phase (Week 0-16 with Week 0 starting at the time of enrollment) using descriptive statistics.

Utilization patterns are reported by demographic characteristics of age group (35-45, 45-55, 65-75 years), gender (male, female), self-identified ethnicity (using a prioritized order as follows: Māori, Pacific, or non-Māori/non-Pacific), and study group (diabetes/prediabetes). Diabetes/prediabetes status was defined using HbA_1c_ levels measured at the start of the study (diabetes range: 50-70 mmol/mol; prediabetes range: 41-49 mmol/mol). Utilization analyses were conducted and plotted in R3.5 (R Institute, Vienna, Austria).

### Participant Perceptions

#### Online Survey

An online questionnaire was completed within REDCap (Research Electronic Data Capture, an electronic data capture tool hosted at the University of Otago [24,25]) by intervention arm participants during the RCT’s final study assessment at 12 months after enrollment. Participants were asked about the usefulness of the full 12-month program and whether they would recommend the BetaMe/Melon program to others. Responses were collected using a 5-point Likert Scale (strongly disagree, disagree, neutral, agree, strongly agree). Data from the questionnaire were extracted from REDCap, analyzed in Microsoft Excel, and reported with descriptive statistics.

#### Participant Interviews

Interview participants were purposefully sampled to highlight Māori and Pacific peoples’ views, with all Māori participants (n=9) and all Pacific participants (n=5) who agreed to be re-contacted invited to interview. The 18 interviews that were completed included all Māori and all Pacific participants who consented and a random sample of participants of other ethnicities stratified by primary care practice. An information sheet and consent form were emailed to all consenting participants prior to the interview.

Telephone interviews were conducted by a researcher with extensive experience in qualitative research (JSt). A semistructured interview schedule (see Multimedia Appendix 1) was used to elicit in-depth participant perceptions about the most and least useful components of the 12-month program, any barriers to incorporating BetaMe/Melon into daily life, and suggested changes to the program. All interviews were recorded digitally.

Participant interview data were analyzed thematically using a primarily inductive approach (led by JSt in close collaboration with VS). First, all interviews were listened to several times so as to familiarize analysists with the data. Second, a summary of findings for each participant was entered into an Excel spreadsheet by interview question. Third, interesting and important data features were coded and a coding framework developed. Fourth, initial themes were developed and reviewed against the dataset. Finally, findings and issues were discussed with other research team members and themes defined and named.

## Results

### Utilization Patterns

Of the 215 intervention participants, 92% (n=198) received an initial health coaching session. Active engagement (at any point in the 16-week monitored period) is shown in Table 1. Of the 215 participants, 160 (74%, 95% CI 68.0-80.1) were actively engaged with the online program components at some point during the 16-week active support phase; the remaining 55 participants (26%) did not actively engage with the online components at any time during the 16 weeks.

Patterns of any engagement in the 16-week period were broadly similar across subgroups. Although there was some variation between groups, much of this variation may be explained by relatively small sample sizes in subgroups rather than representing systematic tendencies for one type of participant to engage more than others (as seen with the CIs for proportions and their absolute differences in Table 1). The exception was engagement by gender, where women were more likely to actively engage at any time (82.4%, 89/108) than men (66.4%, 71/107). For other key comparisons, the differences were inconclusive.

**Table 1:** Patterns of any engagement (at any time in program) by key participant characteristics.

Participant characteristics		Total (N=215), n	Any engagement^a^, n (%), 95% CI	Absolute difference^b^ (95% CI)
**Age (years)**				
	35-54	43	34 (79.1), 64.0-90.0	9.3 (–6.6-25.3)
	55-64	76	53 (69.7), 58.1-79.8	Reference
	65-74	96	73 (76.0), 66.3-84.2	6.3 (–7.1-19.7)
**Ethnicity**				
	Māori	32	21 (65.6), 46.8-81.4	–10.1 (–27.7-7.5)
	Pacific	6	5 (83.3), 35.9-99.6	7.6 (–22.9-38.1)
	Non-Māori / non-Pacific	177	5 (75.7), 68.7-81.8	Reference
**Gender**				
	Male	108	89 (82.4), 73.9-89.1	16.0 (4.6-27.5)
	Female	107	71 (66.4), 56.6-75.2	Reference
**Diabetes status**				
	Prediabetes	105	82 (78.1), 9.0-85.6	Reference
	Diabetes	110	78 (70.9), 61.5-79.2	–7.2 (–18.8-4.4)

^a^Defined as at least one active engagement on the online portal, at any time.

^b^Absolute difference in proportion engaged relative to reference group

**Figure 2:**
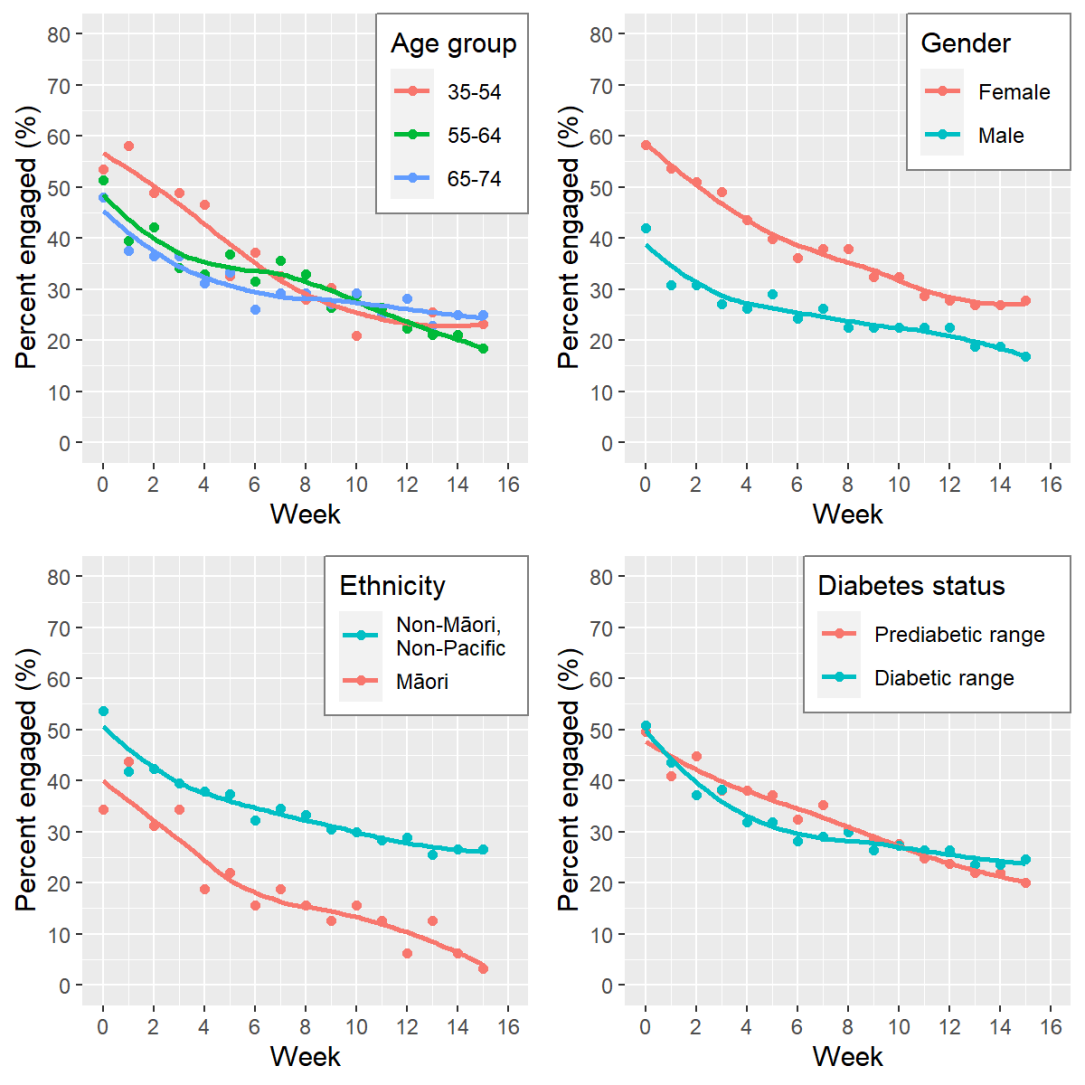
Participant active engagement with BetaMe/Melon portal over time, according to key participant characteristics (age group, gender, ethnicity*, and diabetes status group). *Results for Pacific participants are not presented due to small sample size (n=6).

**Figure 3:**
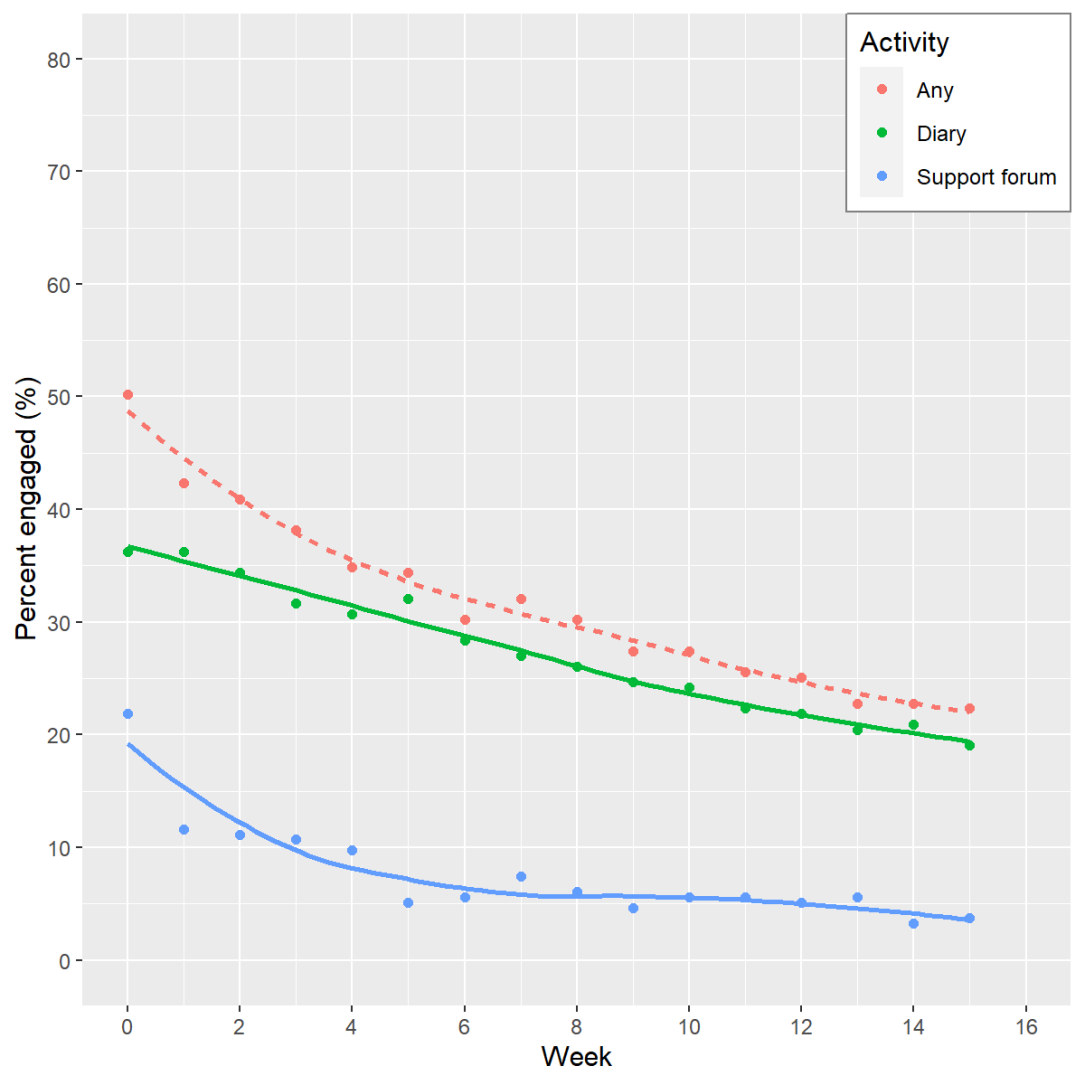
Trends in engagement by week according to type of activity (diary, online peer support forum). “Any” activity represents percentage with any active engagement in that week.

Overall, there was a steady decline in utilization rates (proportion of enrolled individuals actively engaged with the online program in each week) over the 16-week active support phase, from 50% (108/215) of participants engaging at Week 0 down to 22.3% (48/215) at Week 15. Figure 2 shows usage patterns over time by age group, gender, ethnicity, and diabetes status, with all groups showing higher engagement at Week 0 with a steady decline during active program delivery (Weeks 0-15). Participants in the youngest age group (35-54 years) had higher engagement than other age groups, but the trajectory for this age group quickly (by around Week 6) converged with engagement rates for other age groups. Female follow-up engagement was higher, and the percentage of non-Māori/non-Pacific participants engaged at any time point was higher compared to Māori participants. Engagement by participants identifying as Māori decreased the most rapidly to only 3% (1/32) engaging at the end of the 16-week period compared to 27% of non-Māori/non-Pacific participants (47/177). Engagement trajectories were similar for participants with initial HbA_1c_ in the diabetes and prediabetes ranges, following the overall pattern of results. Diary entries (eg, tracking progress toward health goals or changes in weight) were the most frequent form of engagement by all participants (Figure 3).

### Participant Perceptions

#### Online Survey

**Figure 4:**
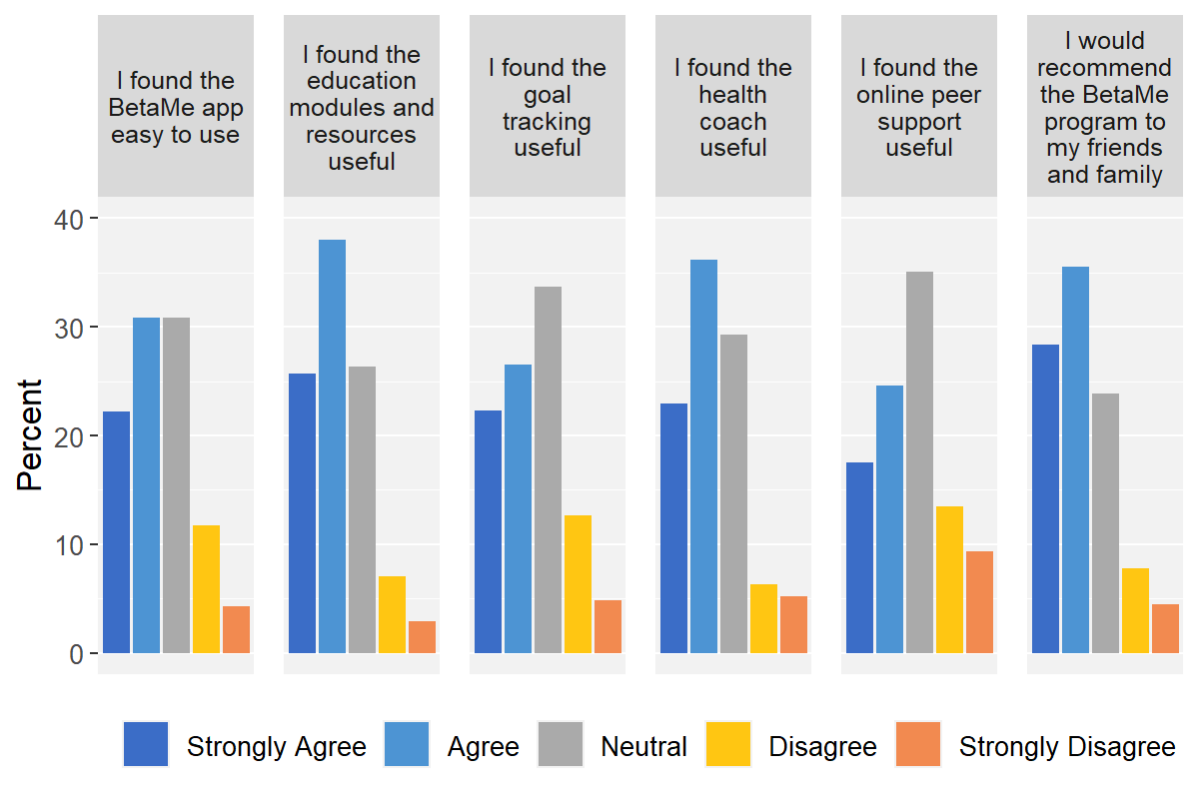
Individual participant feedback from the online questionnaire (percent giving specific response to each item).

The online questionnaire on the usefulness of the entire BetaMe/Melon program (Figure 4) was completed by 83.7% (180/215) intervention arm participants during the final RCT assessment at 12 months. However, not all participants completed all items. Overall, participants rated the education resources and health coaches as more useful components of the program (63.7% [109/171] and 59.2% [103/174] “agreed” or “strongly agreed” with these statements, respectively). Participants considered goal tracking and the online peer support less useful (48.8% [81/166] and 42.1% [72/171] “agreed” or “strongly agreed” with these statements, respectively). About half of respondents (53.0%, 86/162) “agreed” or “strongly agreed” that the program was easy to use. The majority of participants (63.9%, 115/180) indicated that they would recommend the program to friends and family.

#### Participant Interviews

Of the 18 participants who participated in the telephone interviews, 10 were female. Most participants were aged between 45 and 74 years (n=1 aged 35-44, n=10 aged 45-64, n=7 aged 65-74). The majority (n=10) were of non-Pacific/non-Māori ethnicity, 5 were Māori, and 3 were Pacific. Eleven of the 18 participants had HbA_1c_ levels in the diabetes range and 7 had HbA_1c_ levels in the prediabetes range. Interviews lasted 10 to 30 minutes (mean=18 minutes). Thematic analysis of the interviews resulted in four themes about the 12-month program, each of which are described in the following sections and exemplified in Table 2.

**Table 2:** Coding framework extract with example categories and codes.

Theme		Example codes
**Overall program utility**		
	Psychological experience	Good and helpful, helped establish change, improved coping with diabetes, liked being monitored, disliked misinformation about program, lack of attention led to regression
	Technological experience	Program easy to access, trouble with video conferencing, easy, could not access component
	Physical experience	Reduced amount of food eaten, made small changes
**Health coach**		
	Psychological experience	Bubbly, helpful, responsive, easy to understand, supportive, shared personal experiences, motivating, related to coach of same ethnicity, good reinforcement.
	Suggested improvements	Coach continuity, increased coach contact, coaches that are role models (eg, same gender, similar age), continuity of contact, improved follow-up
**Least useful components**		
	Online peer support use	Did not use, did not interact, did not know about this component, read some, not needed, could not access
	Online peer support psychological experience	Not comfortable with medium, did not like, worked well, influential, could not relate to others
	Goal tracking use	Did own tracking, set own goals, easy, did not know about it, did not use it
	Goal tracking psychological experience	Good, helpful, useful, loved it, unsure, unenthusiastic

#### Overall Program Utility

Interview participants found the BetaMe/Melon program to be useful overall, with most indicating that they had a positive experience and identifying beneficial outcomes from participating. For example, a number reported that the program increased their knowledge and led to behavioral changes that helped them lose weight and better manage their diabetes or prediabetes.

Being made more aware of my eating habits. Because being Māori Chinese, when we were little kids we were always told, don’t waste your food, don’t waste your food, so therefore I kept on eating my food, eating my food. And I had a whole sort of regime where I would eat everything on my plate, don’t waste the food.

Participant #10

Most participants reported feeling motivated when they commenced the program and were able to identify components of the program that helped to maintain their motivation. These components included regular contact with the health coach, medication reminders, and the ability to track results relevant to their goals (eg, blood sugar regulation, weight management, and exercising).

The things I did find useful were the reminders to take your medication, I found them very helpful especially the night one cause I wasn’t used to taking that.

Participant #13

The majority of participants reported that they would recommend the program to others.

#### Participant Feedback Regarding Health Coach Element

Connecting with the health coach was the most useful component identified. Participants articulated that regular contact with the online health coaches gave them someone to be accountable to and helped them remain goal oriented. The health coaches were reported as being good communicators, encouraging, positive, and willing to connect with participants on a personal level. One participant indicated that they were so happy with their coach that they wanted to maintain contact following the 16-week active support component of the program.

Very good, very helpful, always there if you needed advice, they were always on the end of the email, so from that point of view they were very, very effective.

Participant #4

However, a number of participants had difficulties connecting or staying connected to their health coach, with one participant explicitly stating they would have preferred a relatable health coach, such as a coach who had been through a weight loss journey and thus would be a role model to aspire to.

Well I’d prefer a female, and someone around my… age I can relate to, or ish, who’s been there and done that, that I can like sort of aspire to like, yay she’s done it maybe I can do it to.

Participant #18

#### Online Peer Support and Goal Tracking

Participants found the online peer support and goal tracking to be the least useful components. Over half of interviewed participants did not use the online peer support component, with personal preference being the primary reason. One participant had technical difficulties accessing the peer support and another reported not knowing about the peer support component of BetaMe/Melon. Three participants read forum posts from time to time but interacted minimally online. Another stopped using the peer support forum, stating that she was unable to relate to other participants due to differences in affordable food options, as what they talked about did not match her budget.

I did put in a comment, you know, has anybody got ideas with pigs head, because that’s just the type of meat I can afford….It wasn’t that beneficial for me, because I was looking at cheap meats, and they were looking at meats I don’t look at….I felt like I was the poor one in the group because my questions were so to the left of what everyone else was on.

Participant #16

The goal tracking component was used by 7 of the interviewed participants, all of whom stated that they found it helpful. Another 5 participants tracked progress against their goals but did not use the BetaMe/Melon program to do so. Six participants did not track their progress against goals at all, although this group included 2 participants who were not aware of the goal tracking component in the program.

I kept getting reminders saying would you input your data, you know your blood sugars, but I didn’t know how to do that or what to do so I never did it. But I keep my own records you know.

Participant #13

#### Barriers and Recommendations

Participants identified a range of problems with the program as well as barriers to its use in daily life. These included problems with the functionality of the program or with internet connectivity; incomplete delivery of all program components for some participants (eg, not setting goals with the health coach or not being informed about all components); and barriers related to the skills, knowledge, or motivation of participants.

Participants suggested a range of improvements to the BetaMe/Melon Program. Suggestions included better support and training regarding how to load and use each component of the program, functionality improvements (such as enabling participants to record specific types of exercise undertaken or share goal tracking information with other willing participants), more frequent and longer contact with the health coach, face-to-face rather than online health coaching, keeping the same coach throughout the program, and use of coaches who are relatable role models. The need to recognize that each participant was unique was commonly suggested. For example, participants suggested efficacy would be improved if BetaMe/Melon were to consider participants’ prior experiences and relative states of minds when commencing the program, including participants’ experiences with goal tracking and weight loss programs, their levels of motivation, and known enablers and barriers to self-management of health conditions. Other suggestions for individualization included increasing program flexibility in order to adapt to participants’ changing life circumstances (eg, being on holiday, unwell, or hospitalized) and tailoring program information and resources to individual participants (eg, providing recipes to cater for those with limited resources).

Suggestions regarding the online peer support included considering other ways of providing this component such as connecting participants so they could meet to share experiences, motivate each other, and participate in group exercise or walks. Participants also suggested that family be included in the program and that a counselling feature be implemented. Finally, participants recommended that the program provide advice on where to go to for help upon completion of the program.

## Discussion

### Overview

This process evaluation was part of a wider study including an RCT [15,26]. It utilized data from 3 sources to assess engagement with and usefulness of a comprehensive digital health intervention program. Overall, 92% (198/215) of intervention participants completed the compulsory first health coach session, although engagement with other components of the program varied by age, ethnicity, and gender and fell over time. Despite this, 64% of respondents (115/180) of participants indicated that they would recommend the program to others, and the majority of those taking part in qualitative interviews following the study period indicated that they found the program to be useful overall.

### Principal Findings

Engagement with the health coach for the first nonoptional coaching session was high at 92% (198/215). Active engagement with other online components of the program peaked in Week 0 with 50% (108/215) of participants actively engaging online. Similar to other programs, participant engagement with the BetaMe/Melon digital health intervention program varied by demographic, with women, younger participants, and ethnic majority population groups demonstrating higher engagement [27,28]. However, engagement dropped steadily over time [28-30]. Reduced engagement over time is a consistent issue with digital health intervention programs [28] and is likely to relate to the attenuation of any health gains achieved by an intervention over time [27,31]. Improving the design of digital health intervention programs to maintain and increase engagement (eg, offering regular rewards, introducing new content to sustain interest, or emailing engagement reminders) and involving specific groups for which the program is likely to be less successful at engaging (either initially or over time) in program development may improve engagement and intervention success [12,28]. 

We found lower overall active engagement with the online components of the program for Māori and Pacific participants. The BetaMe/Melon program was developed using behavioral change and cognitive behavioral theory [30] and included input from Māori and Pacific health care providers. Given the higher prevalence of diabetes and prediabetes in the Māori and Pacific populations of New Zealand, it may be useful for the BetaMe/Melon program (or other digital health intervention programs in New Zealand) to consider using relevant models and behavior change theory specific to Māori and Pacific peoples [32,33]. Internationally, focusing on ethnically appropriate theory when developing and refining digital health interventions may improve engagement and impact for indigenous and minority population groups [31,33,34].

Multimodal programs like BetaMe/Melon have been shown in some studies to be more successful than those adopting a single mode [9,21], with other studies showing that the success of multimodal digital health intervention programs can be moderated by the impact of diabetes self-management style on engagement with and utilization of various components [35]. In general, the BetaMe/Melon program was well received, with educational resources and health coaching seen as the most useful components. However, there was a strong call by participants to strengthen the human components of the program or, in other words, to implement both longer or face-to-face contact with the health coach as well as enhanced peer support activities. Conversely, goal tracking was identified as the least useful component of BetaMe/Melon by participants in both the online survey and telephone interviews. However, diary entries to track progress toward health goals were the most utilized form of engagement irrespective of participant characteristics. Additionally, it appears that some participants were unaware of all components of the BetaMe/Melon program (goal tracking and peer support forum). A few barriers to incorporating the program in everyday life were identified, and several improvements proposed. In particular, the need for increased individualization of the program, a bigger role for the health coaches, and technical improvements to the online components of the program to improve usability were emphasized. Other studies have also called for digital health intervention programs to be tailored to individual participants’ needs [9,12,28,35], to combine digital and human support in order to increase engagement [12,36], and particularly to enable feedback loops between the participant and their health care team in order to improve diabetes control [1,9,12].

### Strengths and Limitations

A key strength of this study is that it was conducted in the context of an RCT of the comprehensive BetaMe/Melon digital health intervention program. We have addressed knowledge gaps around comprehensive self-management programs for people with diabetes and prediabetes and for Māori and Pacific peoples. Another strength is that we were able to describe utilization over time and by type of activity and demographic characteristics (age, ethnicity, gender, and diabetic status).

This study also has some limitations. First, in assessing online engagement with the study, we were limited to the data released by Melon Health Ltd to the Otago University study team. From the data that were provided, we were able to identify the number of completed first health coaching sessions and active engagement with the online program (diary entries and use of the peer support forum). Melon Health Ltd did not supply data on the number or length of health coach sessions per participant or other online activity such as login activity or passive engagement with the program (eg, reading posts or accessing educational material). Second, not all eligible participants answered the online questionnaire, and the participants that agreed to talk with us following the completion of the program may not be representative of the entire study population. Finally, participants were asked 12 months after enrollment to recall their perceptions of a program with a primary focus on the first 16 weeks of delivery. Thus, there is potential for inaccurate recall by participants, and generalizing the findings beyond this study may need careful consideration.

### Conclusions

The BetaMe/Melon program was generally well received by participants, and initial engagement with the health coach was high, although engagement with other components varied by participant demographic and dropped rapidly over the 16-week active program period. Maintaining engagement of participants over time, individualizing programs, and addressing technical barriers to engagement are important to address in order to maximize satisfaction, engagement, and potential health benefits that may come from digital programs for self-management of diabetes.

## Appendix

Multimedia Appendix 1 BetaMe/Melon Process Evaluation Telephone Interview Schedule.

## Abbreviations

HbA_1c_: hemoglobin A_1c_
RCT: randomized controlled trial
REDCap: Research Electronic Data Capture
