# Supplementary material for: A Mobile- and Web-Based Health Intervention Program for Diabetes and Prediabetes Self-Management (BetaMe/Melon): Process Evaluation Following a Randomized Controlled Trial
Source: J Med Internet Res. 2020 Dec 1;22(12):e19150. doi: 10.2196/19150 (PMC7738254; doi:10.2196/19150)
Supplement: Multimedia Appendix 1 [file jmir_v22i12e19150_app1.pdf]

BetaMe/Melon Process Evaluation Telephone Interview Schedule

This is a Multimedia Appendix to a full manuscript published in the J Med Internet Res. For full copyright and citation information see http://dx.doi.org/10.2196/jmir.19150

1. Please tell us about your initial experience of being contacted by the BetaMe/Melon coach and introduction to the program

2. Please tell us how you found the BetaMe/Melon program generally

   Prompts: Impact on everyday life, Changes to food consumption, Changes to weight, Changes to activity, What was positive/good, What could be improved

3. Was the BetaMe/Melon program easy to access? If no, please describe.

   Can you tell us if you think the BetaMe/Melon program has helped you to better manage your diabetes/pre-diabetes? If not, please tell us why?

   Prompts: Skills, Knowledge, Ability

4. Thinking about the different components of the BetaMe/Melon program. Can you tell us your thoughts of
   a. Health coaches - Positive/good, what could be improved
   b. Health modules/resources - Positive/good, what could be improved
   c. Goal tracking - Positive/good, what could be improved
   d. Online peer support - Positive/good, what could be improved

5. The BetaMe/Melon program is delivered in two parts: Core = first 16 weeks/4 months and then core and maintenance for the remaining 36 weeks/8 months
   a. Tell us your thoughts about the length of the 2 components of the program

   Prompts: too short, too long…ask why

   b. Tell us your thoughts about the core program and the maintenance program

6. Were there any gaps in the program? If yes, please describe.

7. Can you describe what other diabetes related support you may have received while you were on the BetaMe/Melon program?

   Prompts: Support from employer, family/whanau/community/GP/ Māori health provider/Pacific health provider

8. Thinking about your participation on the BetaMe/Melon program, can you tell us if you think it may have affected those people close to you

   Prompts: e.g. friends, family, work colleagues, affected exercise or food quality

9. Would you recommend the BetaMe/Melon program to friends/family? Tell us why?

10. Given the experiences and thoughts you've shared with us, what changes, if any, would you suggest be made to the BetaMe/Melon program?

11. Do you have any further comments or questions?
